# The Influence of Arsenic Co-Exposure in a Model of Alcohol-Induced Neurodegeneration in C57BL/6J Mice

**DOI:** 10.3390/brainsci13121633

**Published:** 2023-11-24

**Authors:** Tori R. Sides, James C. Nelson, Kala N. Nwachukwu, Jhana Boston, S. Alex Marshall

**Affiliations:** 1 Department of Biological & Biomedical Sciences, North Carolina Central University, Durham, NC 27707, USA; tsides@eagles.nccu.edu (T.R.S.); jnelso57@nccu.edu (J.C.N.); knwachuk@eagles.nccu.edu (K.N.N.); jboston4@eagles.nccu.edu (J.B.); 2 Integrated Biosciences PhD Program, North Carolina Central University, Durham, NC 27707, USA

**Keywords:** alcohol-related brain damage, hippocampus, arsenic toxicity, microglial activation, CYP2E1, neuroimmune

## Abstract

Both excessive alcohol consumption and exposure to high levels of arsenic can lead to neurodegeneration, especially in the hippocampus. Co-exposure to arsenic and alcohol can occur because an individual with an Alcohol Use Disorder (AUD) is exposed to arsenic in their drinking water or food or because of arsenic found directly in alcoholic beverages. This study aims to determine if co-exposure to alcohol and arsenic leads to worse outcomes in neurodegeneration and associated mechanisms that could lead to cell death. To study this, mice were exposed to a 10-day gavage model of alcohol-induced neurodegeneration with varying doses of arsenic (0, 0.005, 2.5, or 10 mg/kg). The following were examined after the last dose of ethanol: (1) microglia activation assessed via immunohistochemical detection of Iba-1, (2) reactive oxygen and nitrogen species (ROS/RNS) using a colorimetric assay, (3) neurodegeneration using Fluoro-Jade^®^ C staining (FJC), and 4) arsenic absorption using ICP-MS. After exposure, there was an additive effect of the highest dose of arsenic (10 mg/kg) in the dentate gyrus of alcohol-induced FJC+ cells. This additional cell loss may have been due to the observed increase in microglial reactivity or increased arsenic absorption following co-exposure to ethanol and arsenic. The data also showed that arsenic caused an increase in CYP2E1 expression and ROS/RNS production in the hippocampus which could have independently contributed to increased neurodegeneration. Altogether, these findings suggest a potential cyclical impact of co-exposure to arsenic and ethanol as ethanol increases arsenic absorption but arsenic also enhances alcohol’s deleterious effects in the CNS.

## 1. Introduction

Alcohol use disorders (AUDs) are the most prevalent form of substance use disorders, with international estimates suggesting that approximately 100 million people suffer from an AUD [1]. Alcohol is an interesting molecule because it is considered both a drug and a food. Alcohol has psychoactive properties leading to feelings of euphoria, disinhibition, and anxiolysis. However, it is not very potent and requires high doses to be effective. Considering alcohol itself is caloric and often mixed with sweeteners and other fillers, it can make up approximately 16% of the caloric intake for those who drink alcohol [2,3]. One of the consequences of excessive alcohol consumption can be alcohol-related brain damage as well as an increased likelihood of developing other neurodegenerative disorders [4,5]. Both clinical and preclinical evidence suggest that the hippocampus is particularly vulnerable to insults from chronic binge drinking of alcohol [6,7]. Likewise, the hippocampus is susceptible to exposure to high concentrations of arsenic leading to cellular damage and cognitive issues [8,9]. Research has shown that there is a statistically significant relationship between the price of alcoholic beverages and their arsenic concentrations, with less expensive wines containing greater concentrations of arsenic [10]. Presumably, individuals of lower socio-economic status (SES) would be more likely to consume lower-priced, arsenic-contaminated wine. Moreover, arsenic may also be introduced to alcoholic beverages due to processing conditions [11,12]. While the concentration of arsenic in wine may be slight [10], arsenic exposure can be more directly associated with SES as individuals with private wells and less restrictive water purification processes are more apt to be exposed to problematic levels of arsenic [13,14]. The US Environmental Protection Agency (EPA) has issued a standard that arsenic levels should not exceed 10 μg/L or 10 ppb in drinking water. In the US, it is estimated that there are approximately 2.1 million people whose drinking water exceeds the recommended 10 ppb of arsenic levels, sourced from domestic wells [15]. Moreover, arsenic is consistently one of the top three metals found in the soil to exceed US EPA guidelines [16,17]. The prevalence of arsenic toxicity is even more of an issue internationally, with estimates suggesting that approximately 230 million people are exposed to problematic arsenic levels [18]. Several studies have been performed, examining the co-exposure of lead and alcohol, but the influence of arsenic has been less elucidated. Considering the prevalence of excessive alcohol and arsenic toxicity [19,20,21,22,23], the potential co-exposure, and the impact of both on hippocampal integrity, this study seeks to determine whether alcohol and arsenic combine to exacerbate neurodegeneration, microglial activation, and oxidative stress.

More specifically, this study examines reactive oxygen and nitrogen species (ROS/RNS) production and microglial activation as two potentially shared mechanisms of neurodegeneration in these pathologies [24]. Both excessive alcohol consumption [25,26] and arsenic exposure [27,28] can induce neuroinflammatory responses including microglial activation, increases in immune receptors, and up-regulation of proinflammatory cytokines. In this study, Iba-1 was used as an indicator of microglial activity to study the neuroimmune response [26,29,30]. Likewise, oxidative stress is associated with AUDs [31,32] and arsenic exposure [33,34,35], so ROS/RNS levels were assessed using colorimetric assays. Moreover, ROS/RNS production increases after both excessive ethanol exposure and arsenic toxicity have been associated with dysregulation of cytochrome P450s concentrations and activity [33,36,37,38,39,40], so CYP2E1 was also measured in this study as an indicator of ROS/RN production.

The levels of alcohol- and arsenic-induced neurodegeneration are also tied to the concentrations of these toxins in the system. Interestingly, there appears to be some crosstalk between these two toxins as they may directly influence the pharmacokinetic properties of each other. In fact, it has previously been shown that ethanol exposure can increase the arsenic concentrations in the liver preclinical models of AUDs leading to greater hepatoxicity [41,42]. However, the current study seeks to determine if there are regional differences in arsenic absorption by examining the hippocampus whose integrity may be potentially impacted by concurrent alcohol consumption. Likewise, interactions between arsenic and alcohol can impact glucogenic metabolic pathways [43], but little is known about how alcohol and arsenic co-exposure may impact alcohol metabolism. To understand potential interactions in metabolism, this study determined blood ethanol concentrations as well as the concentrations of the alcohol-metabolizing enzyme CYP2E1. CYP2E1 was chosen as previous studies suggest arsenic may impact cytochrome P450s [36], and CYP2E1, as previously discussed, is closely associated with ROS production at higher BECs [44].

Using a rodent model of an AUD associated with neurodegeneration, this study seeks to determine whether arsenic influences alcohol-related brain damage as well as which of the shared mechanisms (microglial activation and ROS/RNS production) may influence cellular damage. Finally, the potential interactions between alcohol and arsenic on each other’s pharmacokinetic parameters will be determined.

## 2. Materials and Methods

### 2.1. Animals

Male and female 7-week-old C57BL/6J mice (*N* = 134; Jackson Laboratories; Bar Harbor, ME, USA) were group housed by sex (2–3 per cage) and maintained in a vivarium (12:12 h light: dark cycle maintained at 22 °C). All animals were given at least a week to acclimate to the environment before experimentation. Throughout these experiments, animals had ad libitum access to food and water. The procedures used in this study were all approved by the North Carolina Central University Animal Care and Use Committee (SAM-11-22-2019) and followed the Guidelines for the Care and Use of Laboratory Animals.

### 2.2. Ethanol and Arsenic Exposure 

Both female and male C57BL/6J received intragastric gavage of ethanol (25% *v*/*v*, Decon Labs, Inc., King of Prussia, PA, USA; CAS No: 64-17-5) or isovolumetric water once daily, for ten consecutive days. The 10-day treatment of 5 g of ethanol/kg body weight was chosen as multiple studies indicate that this dose and duration of ethanol causes neuronal cell loss [45,46,47]. The ethanol and water were adulterated with different concentrations of sodium meta-arsenite (Sigma-Aldrich Co., Saint Louis, MO, USA; CAS No: 7784-46-5). The highest dose of arsenic (10 mg/kg) was chosen because it independently leads to neurodegeneration after 10 days, but the middle dose (2.5 mg/kg) did not lead to neuronal damage [8,48]; however, the lowest dose, 0.005 mg/kg arsenic, was based on the US EPA standards [49]. See Table 1 for additional details concerning the animal number and treatment for each subgroup. 

For immunohistochemistry studies, the brains of mice (*N* = 66) were processed similarly to our previous studies [50,51]. Briefly, mice were euthanized by transcardial perfusion for approximately 30 min but within 1 h of the final dose of ethanol. Mice were anesthetized via IP administration (~0.1 mL) of a ketamine/xylazine cocktail (66.7 mg/mL; 6.7 mg/mL). PBS was flushed through for approximately 5 min followed by 4 min of 4% paraformaldehyde (PFA). The brain was extracted and stored in 4% PFA solution for 24 h then changed out to be stored in cryopreserve solution (ethylene glycol, Polyvinyl-pyrrolidone, and 0.2 M Phosphate Buffer; pH = 7.4). The brains were sectioned coronally at 40 μm with a vibrating microtome (Compresstome^®^ VF-310-0Z, Precisionary Instruments Inc.; Greenville, NC, USA) and stored in cryopreserve in a 1:4 series until further processing. It is important to denote that all groups started with 10 animals. However, 8 animals died prematurely due to gavage errors. This premature loss is within the range (~5–10%) of other studies, but importantly did not appear to be associated with the treatment of the animals as no differences were observed in weights or general health (See Table 2). The best survival rate was actually in animals that were in the highest arsenic exposure group. Six additional animals were removed because there were insufficient sections with the appropriate region for analysis.

For ELISA/ROS (*N* = 28) and ICP-MS studies, mice (*N* = 40) were euthanized by rapid decapitation at approximately 30 min but within 1 h of ethanol exposure. No animals were removed or lost in either the ELISA/ROS assay or ICP-MS cohorts. ICP-MS -The hippocampus was then bilaterally micro-dissected out so that the tissue collected herein represents the hippocampus or brain (minus hippocampus). In addition to brain and hippocampal tissue, liver, and trunk blood samples were collected to measure arsenic, CYP2E1, and/or ROS/RNS concentration. All tissue was snap-frozen on dry ice and stored at −80 °C until further processing. The blood samples were centrifuged for 5 min at 1500× *g* to separate the serum from the red blood cells and immediately stored at −20 °C.

### 2.3. Blood Ethanol Concentration Determination

Similar to our previous publications [25,50], trunk bloods were collected approximately 30 min but within one hour after the final ethanol dose to reflect the peak blood ethanol concentrations [52,53]. BECs (mg/dL) were quantified from the serum using the colorimetric assay, EnzyChrom Ethanol Assay Kit (BioAssay Systems; Hayward, CA, USA). The absorbance was measured using Synergy HTX Microplate Reader (BioTek Instruments, Inc., Winooski, VT, USA). Serum samples were run in duplicates and are presented in mg of ethanol/dL of serum. 

### 2.4. Immunohistochemistry & Immunoreactivity Quantification

Microglial activation (Iba-1) was assessed using immunohistochemistry, as previously described [50,54]. Ionized calcium-binding adaptor molecule 1 (Iba-1) was used because it plays an integral role in the rearrangement of the membrane cytoskeleton, and microglia make dynamic changes to their membrane cytoskeleton as they become more active [29]. For immunohistochemistry, phosphate buffer saline (PBS, pH = 7.4) was used to remove cryopreserve. The wash was performed 3 times for 10 min followed by an incubation in 0.6% H_2_O_2_ for 30 min. Following 3 more washes in PBS for 10 min, a blocking solution of PBS, 0.1% triton X-100, and 3% goat serum (Vector Laboratories, Burlingame, CA, USA) was added to the tissue for 30 min to reduce non-specific binding of the primary antibodies. The tissue was washed and then incubated for 72 h in the well-validated rabbit anti-Iba-1 (Fujifilm Wako Pure Chemical Co., Osaka, Japan; AB_839504) for 72 h at 1:1000 at 4 °C. On the second day, 3 washes of PBS were performed for 10-min each then the tissue was incubated in goat anti-rabbit biotinylated secondary antibody (1:2000) for 1 h. After a series of PBS washes, ABC (avidin-biotin-complex; Vector Laboratories, Burlingame, CA, USA) was used to form a chromogen complex with 3,3′-diaminobenzidine (DAB; ACROS Organics; Morris Plain, NJ, USA). Sections were then washed in PBS, mounted, and coverslipped with Cytoseal™ (Thermo Scientific, Waltham, MA, USA). 

Photomicrographs of the hippocampus were obtained using a 10× objective on a B120 microscope (AmScope; Irvine, CA, USA) with an attached AmScope MU503 digital camera as previously described [51]. To reduce experimenter bias, experimenters were blinded to the treatment groups during quantification, and immunoreactivity was quantified using QuPath 0.2.3, an open-source image analysis program [55,56]. For photomicrographs, the three subregions of the hippocampus (dentate gyrus [DG], cornu amonis [CA]1, and CA2/3) were individually traced on each image, and the percent area of staining was obtained based on experimenter-determined thresholds. At least 5 sections were included for each animal between Bregma −1.06 mm and −2.54 mm [57]. The DAB immunoreactivity for Iba-1 is expressed as a density measure (% area); additionally, cell counts were quantified by automation combining threshold and size to determine cell number (Iba-1+ cells/section). 

### 2.5. Fluoro-Jade C Staining & Quantification

To quantify neurodegeneration, cell counts were conducted following staining with Fluoro-Jade^®^ C (FJC) similar to our previous studies [54,56]. FJC is a fluorochrome that labels degenerating neurons in the CNS [58]. Briefly, sectioned tissue was mounted and dried on slides before processing. Fluoro-Jade^®^ C staining was performed by immersion of the slides in the following solutions: 2 min in 70% ethanol, 2 min in DI water, 10 min in 0.06% potassium permanganate, 2 min in DI water, and then 15 min in the FJC solution (0.001% (*w*/*v*) dissolved in 0.1% (*v*/*v*) acetic acid). Finally, the slides were rinsed through submersion in three changes of distilled water for 1 min each. The air-dried slides were then cleaned in xylene for at least 1 min and then cover slipped using SHUR/Mount™ (Triangle Biomedical Sciences; Durham, NC, USA). FJC+ cells within the hippocampal subregions were quantified at 10× using a fluorescence microscope (Olympus BX51 Fluorescence Microscope; Center Valley, PA, USA) camera and are expressed as cell/section.

### 2.6. CYP2E1 ELISA

Tissue from the hippocampus and liver were collected within an hour of final ethanol gavage and homogenized in an ice-cold lysis buffer (1 mL of buffer/50 mg of tissue; pH = 7.4). The lysis buffer included 25 mM HEPES, 0.1% 3-[(3-cholamidopropyl) dimethyl-ammonio]1-propanesulfonate, 1.3 mM EDTA, 1 mM EGTA, 10 µg/mL aprotinin, 10 µg/mL leupeptin, 5 mM MgCl2 (Fisher, Fairlawn, NJ, USA), 10 µg/mL pepstatin (Fluka, Milwaukee, WI, USA), and 1 mM PMSF [26,59]. Pierce BCA Protein Assay Kit (Thermo Scientific, Waltham, MA, USA) was utilized to determine total protein content according to manufacturer instructions. Protein concentrations were determined from absorbance measured in duplicates at 562 nm on a BioTek plate reader. There was sufficient protein (µ-hippocampus = 540.4 µg/mL; liver = 1268.45 µg/mL) in each sample so there was no need to pool tissue samples for the ELISA. To determine CYP2E1 concentrations, ELISA kits were purchased from My BioSource (San Diego, CA, USA; MBS453581) [60]. Samples were run in duplicate, and the absorbance was compared to standards as read on a BioTek plate reader at 450 nm. CYP2E1 concentrations were normalized to the total protein and are expressed as ng of CYP2E1/mg of protein.

### 2.7. Reactive Oxygen/Nitrogen Species Assay

Total ROS/RNS levels were quantified using the OxiSelect In Vitro ROS/RNS Assay Kit (Cell Biolabs, Inc., San Diego, CA, USA) [61,62,63]. This kit utilizes a fluorogenic probe that reacts with free radicals. Total fluorescence gives an indication of total free radicals in the sample, measured against a hydrogen peroxide standard. Liver and hippocampus tissue samples were homogenized in ice-cold lysis buffer made with 40 mM Tris buffer (pH 8.0) containing 120 mM NaCl, 0.5% NP-40, 100 μM sodium-orthovanadate, 2 μg/mL aprotinin and 5 μg/mL leupeptin [64]. The tissue homogenates were then centrifuged at 10,000× *g* for 5 min to remove insoluble particles. Samples were loaded into 96-well plates in duplicates and the assay was performed in accordance with the kit protocol. ROS/RNS concentrations were determined using a BioTek plate reader at 480 nm excitation/530 nm emission.

### 2.8. Inductively Coupled Plasma Mass Spectrometry & Arsenic Concentrations

Arsenic concentrations were determined from fresh frozen liver, hippocampus, and brain tissue by inductively coupled plasma mass spectrometry (ICP-MS). ICP-MS was performed in collaboration with the Molecular Education, Technology and Research Innovation Center (METRIC) at NC State University similar to previous studies [65,66]. Briefly, samples were digested by heating in 800µL of nitric acid and 400 µL of hydrochloric acid at 95 °C for an hour followed by an hour incubation in 30% H_2_O_2_. Internal standards of germanium, indium, praseodymium, and yttrium were added to the samples and diluted in DI water. A standard solution containing 1 ppb each of Li, Co, In, Ba, Ce, Bi, and U in 2% HNO_3_ and 0.5% HCl was used to tune the mass spectrometer with the automated tuning and performance evaluation routines. The calibration levels used for As were as follows in ppb: 0.01, 0.05, 0.1, 0.5, 1, 5, 10, 50, 100, and 500. Each standard level also contained Ge internal standard at 50 ppb. All calibration points acquired with each set of samples returned calculated values within 30% of the nominal values. The 10 ppb and 50 ppb As standards were used as quality control samples and alternately infused during each automated sequence after 19–21 sample measurements. Calculated ppb values for As were within 30% for all QC measurements. Standard and quality control infusions were bracketed by blank solvent washes. Arsenic content was determined using the iCAP RQ ICP-MS and Qtegra™ Intelligent Scientific Data Solution™ Software 2.8 (ThermoScientific, Waltham, MA, USA). Arsenic concentrations were normalized to the tissue weight and are reported as µg of arsenic/g of tissue. 

### 2.9. Statistics

All data were graphed and analyzed using GraphPad Prism 9.0.1.151. All histology, ELISA, and ROS/RNS assay data were analyzed using two-way analyses of variance (ANOVAs; ethanol treatment × arsenic dose), but ICP-MS and BEC data used one-way ANOVAs to determine statistical significance. The ethanol dose used has previously been shown to increase microglial reactivity, cell death, and pro-inflammatory cytokines in this model, so the goal of the current study was to determine if there was an additive effect of arsenic as well as the dose of arsenic that influences these markers. As such, planned Bonferroni post hoc analyses compared to the water and ethanol alone groups were used if there was an interaction or main effects of both arsenic and ethanol. Comparisons after main effects are warranted when there is an a priori hypothesis such as that of the ethanol treatment reported herein [67]. All data presented in the figures were mean ± SEM. Effects were considered significant if *p* < 0.05.

## 3. Results

### 3.1. Effects of Ethanol & Arsenic on Microglial Activation

**Figure 1:**
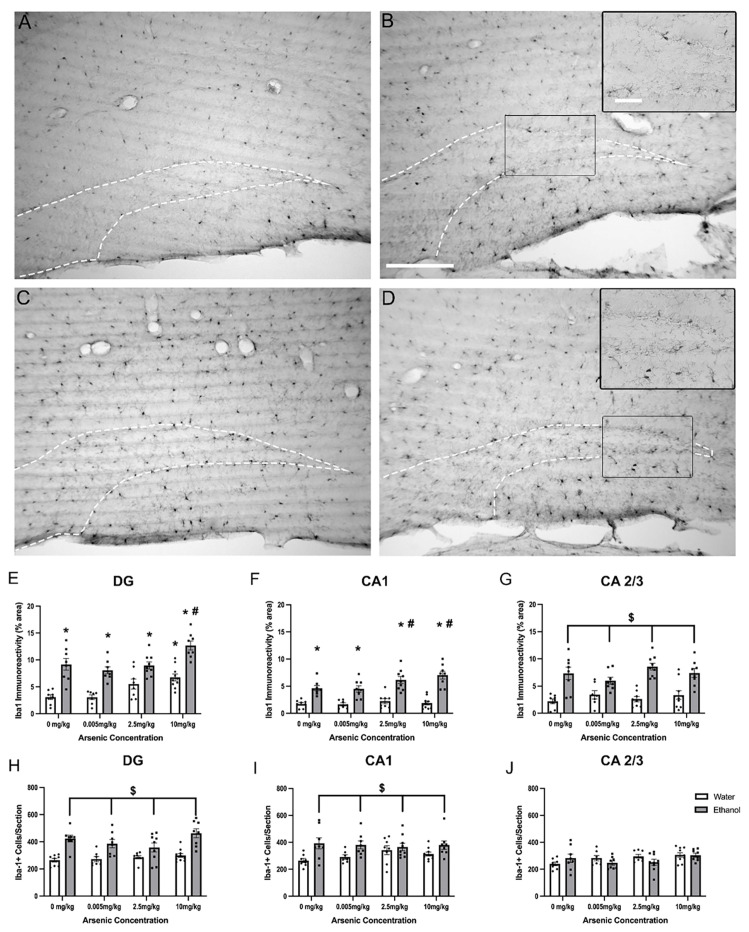
Additive Effects of Ethanol & Arsenic on Microglial Activation. Photomicrographs of the dentate gyrus suggest Ionized calcium-binding adapter molecule 1 (Iba-1) positive cells and immunoreactivity (IR) are increased (hilus outlined by the white dashes) in mice following a daily 10-day dose schedule of 10 mg/kg arsenic (**B**), 5 g/kg ethanol (**C**), ethanol and arsenic combined (**D**) compared to the water group (**A**). Ethanol alone led to an increase in Iba-1 IR throughout the hippocampus (**E**–**G**), but only in the DG (**E**) did the highest dose of arsenic lead to significantly more Iba-1 pixels. Importantly, when the two were combined, there appeared to be an additive response to arsenic compared with the ethanol group in the DG (**E**) and CA1 (**F**). Ethanol also led to an increased number of Iba-1+ cells in the DG (**H**), and CA1 (**I**), but not the CA2/3 subregion (**J**), but arsenic did not seem to have an influence on cell number. Scale bar in (**B**) = 200 μm, inset scale bar = 50 μm (*, *p* < 0.05 compared with water only; #, *p* < 0.05 compared with ethanol only; $, *p* < 0.05 main effect of ethanol; circles represent the individual data points).

Changes in microglia morphology are an indicator of microglia activation. All hippocampal sub-regions expressed Iba-1+ cells; however, qualitatively distinct morphological differences in Iba-1+ cell morphology between control and ethanol mice were seen as shown in representative images of the DG (Figure 1). Quantitative analysis confirmed that both arsenic [F (3, 58) = 12.45, *p* < 0.0001] and ethanol [F (1, 58) = 93.72, *p* < 0.0001] had a main effect on Iba-1 immunoreactivity in the DG but no interaction was determined. Posthoc Bonferroni tests indicate that ethanol increased Iba-1 immunoreactivity in all groups and that the highest concentration of arsenic was significantly different from the water control. Moreover, there was a significant increase in Iba-1 immunoreactivity in the DG comparing ethanol and ethanol with the highest dose of arsenic, suggesting an additive effect of arsenic. In the CA1, a two-way ANOVA showed both arsenic [F (3, 58) = 3.86, *p* = 0.01] and ethanol [F (1, 58) = 109.1, *p* < 0.0001] had a main effect on Iba-1 immunoreactivity but no interaction was determined. Posthoc analyses suggested that ethanol increased microglia reactivity regardless of arsenic concentration, and that despite no observed increase by arsenic alone, arsenic enhanced the ethanol effects at the two highest doses of arsenic. Finally, in the CA2/3 region, a two-way ANOVA [F (1, 58) = 69.48, *p* < 0.001] showed a main effect of ethanol suggesting that ethanol increased microglia reactivity, but no effect of arsenic [F (3, 58) = 0.67, *p* = 0.58] or interaction [F (3, 58) = 1.83, *p* = 0.15] between the variables was determined by two-way ANOVAs. Because immunoreactivity can also be influenced by cell number, Iba-1+ cell counts were estimated using an automated counting system. In the DG and CA1, two-way ANOVAs indicated that ethanol led to an increase in microglia cell number [DG: F (1, 58) = 55.36, *p* < 0.001; CA1:F (1, 58) = 15.61, *p* < 0.001] but no interactions [DG:F (3, 58) = 1.64, *p* = 0.19; CA1:F (3, 58) = 0.34, *p* = 0.80] or main effects of arsenic [DG:F (3, 58) = 2.48, *p* = 0.07; CA1:F (3, 58) = 0.34, *p* = 0.80] were observed. The CA2/3 appeared to be more resilient to ethanol’s effects on cell number as a two-way ANOVA indicated neither a main effect of ethanol [F (1, 58) = 0.51, *p* = 0.48] or arsenic [F (3, 58) = 2.30, *p* = 0.09] and no interaction [F (3, 58) = 2.06, *p* = 0.12].

### 3.2. Arsenic & Ethanol Impacts on CYP2E1 Expression in Liver

**Figure 2:**
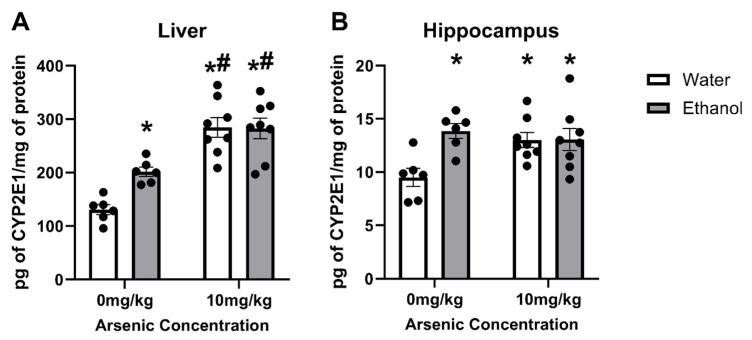
No additive effects of arsenic and ethanol on CYP2E1 expression in the liver or hippocampus. Arsenic (10 mg/kg) and ethanol (5 g/kg) lead to an increase in CYP2E1 concentration in the liver (**A**) and hippocampus (**B**). However, no additive or synergistic effects were observed when ethanol and arsenic were combined. (*, *p* < 0.05 compared with water; #, *p* < 0.05 compared with ethanol; circles represent the individual data points).

Arsenic effects on CYP2E1 could influence both ethanol metabolism and lead to excessive ROS production. As such, ELISAs were used to assess the concentration of CYP2E1. A two-way of CYP2E1 concentrations in liver samples indicated there was an interaction [F (1, 24) = 4.97, *p* = 0.035] as well as main effects of both arsenic [F (1, 24) = 51.28, *p* < 0.0001] and ethanol treatment [F (1, 24) = 26, *p* = 0.045]. Likewise, there was an interaction [F (1, 24) = 6.19, *p* = 0.020] and the main effect of ethanol [F (1, 24) = 6.51, *p* = 0.018] but not arsenic dose [F (1, 24) = 2.48, *p* = 0.13]. Posthoc Bonferroni analyses indicated that in both the liver and the hippocampus arsenic and ethanol led to an increase in CYP2E1 independently, but that no additive effects occurred when ethanol and arsenic were combined. However, the effect of arsenic in the liver induced significantly more CYP2E1 than ethanol alone as seen in Figure 2.

### 3.3. Arsenic Influences on Blood Ethanol Concentrations

**Figure 3:**
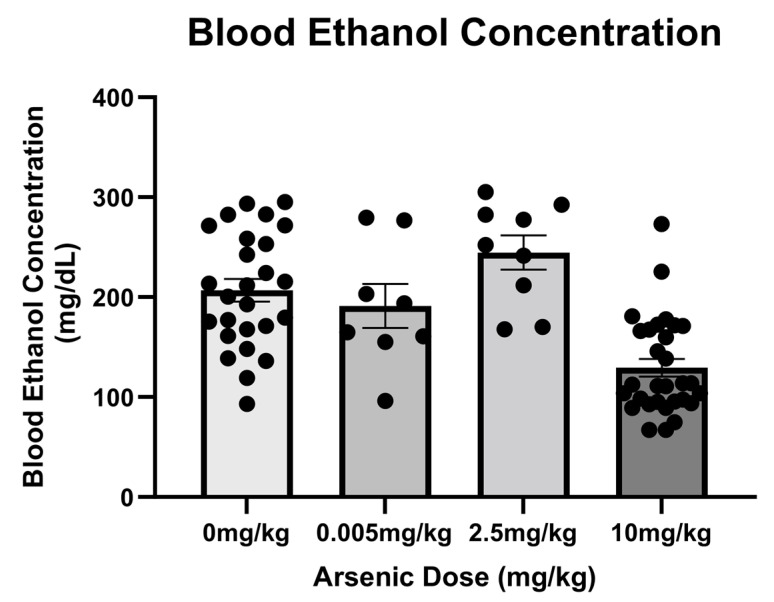
High Doses of Arsenic Increased Ethanol Clearance. The group receiving the highest dose of arsenic (10 mg/kg) had significantly lower BECs compared to other ethanol groups (circles represent the individual data points).

BECs were measured in mice that had received a 5 g/kg dose of ethanol with differing concentrations of arsenic. To simplify analyses, animals with identical treatment were collapsed across all three studies (e.g., immunohistochemistry vs. ELISA) as no differences were observed between cohorts. A one-way ANOVA comparing BECs among treatment groups indicated an effect of arsenic treatment [F (3, 69) = 15.45, *p* < 0.0001]. Posthoc Bonferroni analysis indicated that the group of animals that received the highest dose of arsenic actually had lower BECs than all the other ethanol groups despite receiving the same dose of ethanol (Figure 3). 

### 3.4. Ethanol & Arsenic-Induced ROS/RNS Production

**Figure 4:**
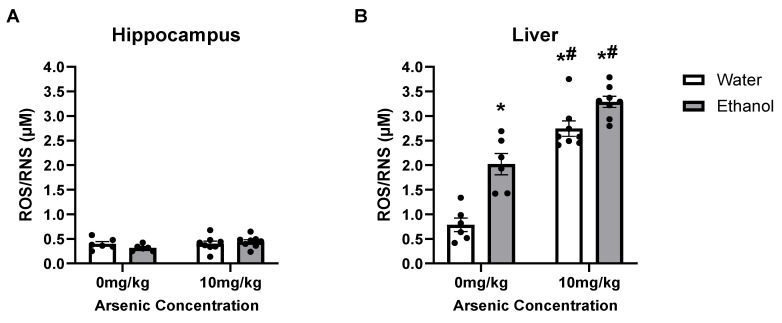
Ethanol had no additive effects on arsenic-induced ROS/RNS production. Arsenic exposure caused an increase in ROS/RNS in the liver (**B**) but not in the hippocampus (**A**), but there were no additive effects of ethanol to arsenic’s effects. (*, *p* < 0.05 compared with water; #, *p* < 0.05 compared with ethanol; circles represent the individual data points).

ROS can be a byproduct of ethanol metabolism but is also associated with toxic levels of arsenic, especially when CYP2E1 is engaged. In the liver (Figure 4B), a two-way ANOVA indicated that there was an interaction [F (1, 24) = 4.82, *p* = 0.038] as well as main effects of both ethanol [F (1, 24) = 31.89, *p* < 0.0001] and arsenic [F (1, 24) = 104.7, *p* < 0.0001]. Posthoc Bonferroni’s tests indicated that arsenic and ethanol both led to an increase in ROS/RNS production compared to the control group, but there was no enhancement when ethanol was included. However, a two-way ANOVA suggested that neither arsenic [F (1, 24) = 2.04, *p* = 0.17] nor ethanol [F (1, 24) = 0.22, *p* = 0.64] caused an increase in ROS/RNS production in the hippocampus as there were no differences observed between any of the groups or any interaction [F (1, 24) = 1.84, *p* = 0.19], as shown in Figure 4A. 

### 3.5. Ethanol Increases Arsenic Absorption in the Liver and Hippocampus

**Figure 5:**
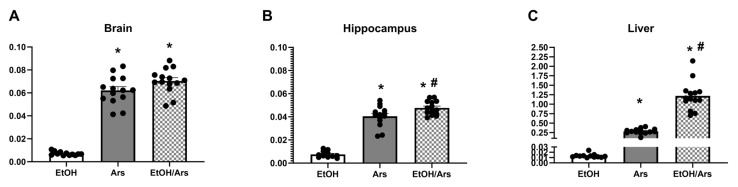
Ethanol increases arsenic absorption in the liver and hippocampus. ICP-MS measurements of arsenic (Ars) concentration showed the arsenic treatment effect was enhanced by ethanol in the hippocampus (**B**) and liver (**C**) but not in the brain (**A**). (*, *p* < 0.05 compared to ethanol alone; #, *p* < 0.05 compared with ethanol; circles represent the individual data points).

ICP-MS was used to measure arsenic concentrations in the liver, brain, and hippocampus to determine if ethanol impacted the pharmacokinetics of arsenic. In the brain, a one-way ANOVA indicated that there was an effect of treatment [F (2, 37) = 155.5, *p* < 0.001]. Posthoc analyses indicated that with the same arsenic dose both ethanol and water groups had similar concentrations of arsenic. Likewise, one-way ANOVAs of arsenic concentration in the liver [F (2, 37) = 155.5, *p* < 0.001] and hippocampus [F (2, 37) = 155.5, *p* < 0.001] indicate that there was an effect of treatment; however, in these areas the arsenic treatment effect was enhanced by ethanol according to posthoc Bonferroni tests as presented in Figure 5.

### 3.6. Regionally Specific Enhancement of FJC+ Cells with Ethanol & Arsenic

**Figure 6:**
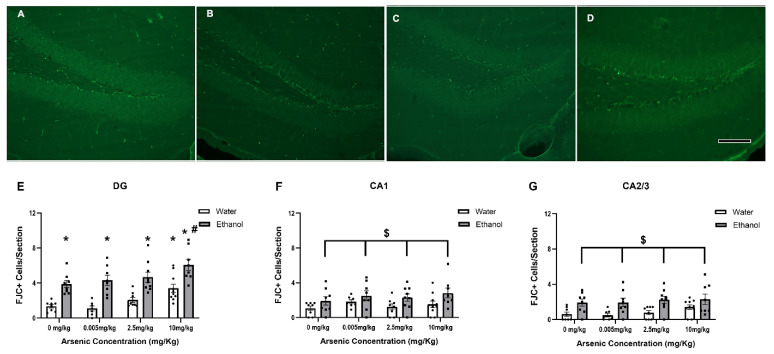
Arsenic enhances ethanol-induced neurodegeneration in the DG. Photomicrographs of FJC-positive cells in the DG suggest that arsenic (**B**) and ethanol (**C**) were associated with more cell death compared with the control (**A**), but this effect was enhanced when arsenic and ethanol were combined (**D**). Quantification of these cells indicated that arsenic led to a more robust response to ethanol in the DG (**E**). However, there was only an effect of ethanol in the CA1 (**F**) and CA2/3 (**G**). Scale bar in (**D**) = 25μm (*, *p* < 0.05 compared with water only; #, *p* < 0.05 compared with ethanol only; $, *p* < 0.05 main effect of ethanol; circles represent the individual data points).

Two-way ANOVAs were performed to determine whether co-exposure to arsenic and excessive ethanol enhance neurodegeneration. In the DG (Figure 6E), both arsenic [F (3, 58) = 8.82, *p* < 0.0001] and ethanol [F (1, 58) = 67.15, *p* < 0.0001] had a main effect on the number of FJC+ cells, but no interaction [F (3, 58) = 0.21, *p* = 0.89] was determined. Posthoc Bonferroni tests indicate that ethanol led to more FJC+ cells at similar levels for the lowest concentrations of arsenic; however, with 10 mg/kg arsenic and ethanol, there was an enhanced effect of ethanol compared to the ethanol alone group. The highest dose of arsenic also increased FJC+ cells independently suggesting the increase in FJC with co-exposure was likely just additive. Moreover, there was a significant increase in FJC+ cells in the CA1 (Figure 6F) and CA2/3 (Figure 6G)regions after ethanol. Two-way ANOVAs indicated a main effect of ethanol (CA1: [F (1, 58) = 10.26, *p* = 0.002], CA2/3: [F (1, 58) = 22.66, *p* < 0.0001]) but no independent effect of arsenic (CA1: [F (3, 58) = 1.15, *p* = 0.34], CA2/3: [F (3, 58) = 1.21, *p* = 0.32]) or any interaction (CA1: [F (3, 58) = 0.19, *p* = 0.90], CA2/3: [F (3, 58) = 0.26, *p* = 0.87]) between the variables for the CA1 and CA2/3, respectively.

## 4. Discussion

**Figure 7:**
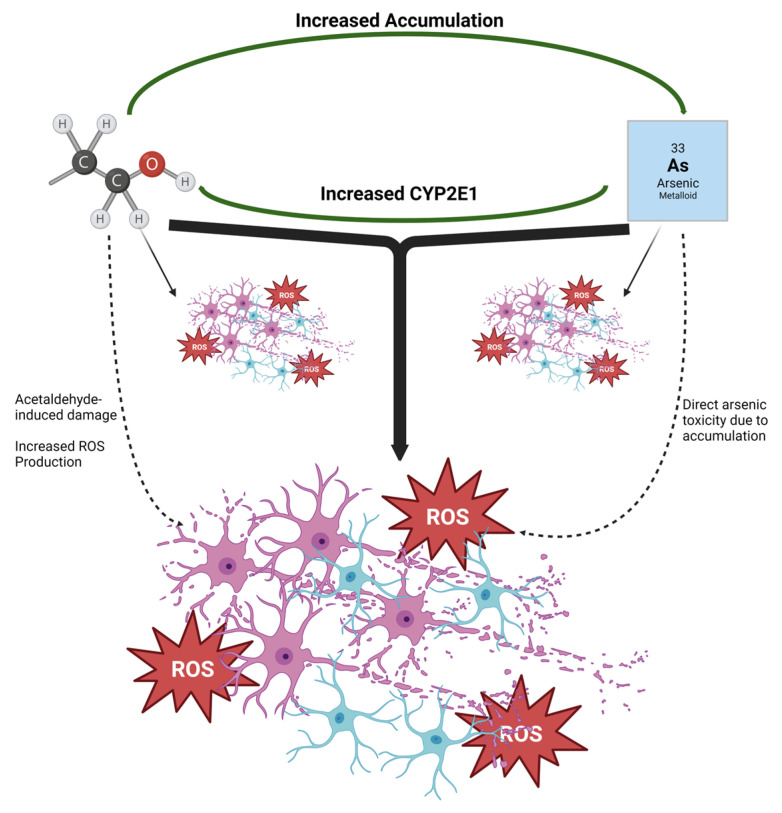
Additive effects of ethanol and arsenic on neurodegeneration. Both ethanol and arsenic led to neurodegeneration, microglial activation, and ROS production with these negative effects being additive with co-exposure. Alcohol increased arsenic accumulation in the hippocampus and liver which may have influenced arsenic toxicity explaining the additive effects of co-exposure. Likewise, the additive effect of co-exposure could be associated with arsenic’s increase in CYP2E1 which had the potential to lead to an increase in both acetaldehyde and ROS production. Solid lines represent the current data whereas the dashed lines are suggestions of potential mechanisms.

Both excessive ethanol and arsenic toxicity can lead to neurodegenerative events. In the case of an AUD or living in areas with higher arsenic levels in drinking water, toxic concentrations and or consumption can be lifelong issues [68,69]. Given the shared mechanisms of neurodegeneration shared by AUDs and arsenic exposure, the purpose of this study was to determine whether the two exacerbated one another leading to any enhanced effects on hippocampal damage. The data presented herein indicate that the co-exposure of ethanol and arsenic leads to (1) enhanced microglial reactivity and neurodegeneration in a regionally specific manner, (2) arsenic impacts ethanol metabolism, and that (3) ethanol increases arsenic absorption. For the purposes of this work, additive responses are defined as an enhanced effect compared to ethanol when there was also an independent effect of arsenic, whereas synergism is defined as an enhanced effect in ethanol despite no effect with arsenic alone. These studies suggest that there may be a potential for combinatorial effects of alcohol and arsenic on neurodegeneration through increased microglial activation and ROS production. These maladaptations could potentially be due to alcohol-induced increases in arsenic absorption or because of arsenic-induced changes in alcohol pharmacokinetics as reviewed in Figure 7.

Alcohol-induced microglial activation is commonly observed across various models of AUDs. In the 10-day gavage model used herein, it has previously been established that there are increases in microglial activation based on morphological presentation as well as increases in proinflammatory cytokines and chemokines [47,70]. It was therefore not surprising that there were increases in Iba-1 immunoreactivity; however, this is the first study to suggest that this 10-day model of alcohol-induced neurodegeneration also leads to an increase in microglial cell number. The increase in Iba-1+ cells suggests that there may be a proliferation of microglia as observed in clinical samples examining a history of alcohol abuse as well as other AUD preclinical models [59,71,72]. However, it is also plausible that the microglia migrate from other areas due to increased susceptibility to alcohol-induced damage in the hippocampus [6,73,74] or that Iba-1+ cells may have been increased due to invading peripheral macrophages [75]. Less clear was whether arsenic would independently cause microglial activation, as the microglial response after the arsenic exposure used had not previously been tested. However, other arsenic toxicity studies have shown mixed results concerning arsenic’s effects on microglia. For example, previous research found that much lower doses of arsenic resulted in microglia that were more responsive to LPS [28] and that chronic arsenic exposure using similar concentrations of arsenic (50 mg/L) resulted in a shift in microglia from an M2 to M1 presentation [76]. However, the current study indicates that a shorter exposure to arsenic also leads to an increase in Iba-1 density in the dentate gyrus. Arsenic did not appear to have any effect on microglial number in agreement with others who observed that arsenic did not influence microglial viability [77]. The increased reactivity of microglia to arsenic resulted in an additive effect in Iba-1 density in the DG when ethanol was also presented. However, in the CA1 and CA2/3, arsenic did not have an independent effect on a measure of microglial reactivity suggesting that the DG may be more susceptible to arsenic’s effects. Despite no independent influence of arsenic on CA1 microglia, an enhanced effect in alcohol-induced increased Iba-1 density was observed at the highest two doses of arsenic suggesting a synergistic relationship. When alcohol and arsenic were combined in the CA2/3, the microglial density was consistent with ethanol alone. Although the effects of alcohol and arsenic co-exposure were somewhat regionally specific, this data suggests that arsenic exacerbates alcohol-induced microglial reactivity.

When alcohol dehydrogenase is overwhelmed by higher BECs, CYP2E1 will metabolize ethanol, but it can lead to higher ROS production and a buildup of the toxin acetaldehyde [44]. Arsenic’s effect on CYP2E1 was measured in liver and hippocampus homogenates. Ethanol and arsenic led to an increase in CYP2E1 in the hippocampus congruent with previous studies [44,78,79], but no differences were observed when ethanol and arsenic were combined. However, in the liver, arsenic caused an increase in CYP2E1 that surpassed the expected ethanol-induction previously reported [80,81], but the arsenic response was not further enhanced with alcohol. Previous research has shown that arsenic alone can alter the expression of the cytochrome P450 enzymes [82], but this is the first study to specifically examine arsenic’s effects on the alcohol-metabolizing enzyme, CYP2E1. Although these studies do not support an additive effect between arsenic and ethanol on CYP2E1, previous research showed that alcohol and arsenic co-exposure leads to the elevation of liver transaminases and other liver enzymes [42]. 

The increase in the CYP2E1 enzyme in the liver after high doses of arsenic may also explain the observed decrease in blood ethanol concentrations. The current observations were limited to BECs, but it is plausible that the increase in ethanol metabolism from the arsenic-induced enzyme changes may have caused a buildup of acetaldehyde [44,83]. The observation of lower BECs in the highest arsenic group supports the idea that there may indeed be a more complicated relationship between arsenic and ethanol pharmacokinetics. The animals in this experiment were gavaged consistent doses of ethanol, but in clinical situations increases in ethanol metabolism have been shown to increase alcohol abuse liability and other pathological consequences [84,85]. The downstream effects of increased ethanol metabolism by arsenic were not tested in the current study, but these data support the idea that toxic levels of arsenic impact the pharmacokinetics of ethanol.

Ethanol metabolism via the CYP2E1 pathway has been tied to oxidative stress [32,44], so the observed arsenic-induced increase in CYP2E1 levels was expected to increase ROS production. In the hippocampus, where arsenic did not alter the CYP2E1 concentration, there were no significant changes in ROS/RNS production even after ethanol consumption alone. Others have reported an increase in oxidative stress in the hippocampus after ethanol exposure using in vitro techniques or indirect measurements [37,86,87]. It is possible that there was a basement effect based on the threshold of the current methodology. However, in the liver, both arsenic and ethanol-induced increases in CYP2E1 aligned with an increase in ROS/RNS. Surprisingly, there was no additional effect on liver ROS/RNS levels in the presence of alcohol. Many studies of alcohol exposure indicate elevated CYP2E1 leads to increased ROS production [37,88], but these studies were a single snapshot during intoxication whereas ROS/RNS production may be elevated over a time course [86,89,90]. 

The current work aligns with clinical findings that a history of excessive alcohol consumption can increase the levels of various metals in AUD patients [91,92]. More specifically, these data indicate that ethanol consumption caused an increase in arsenic levels in the hippocampus and liver. The increase in the arsenic levels may be due to increased absorption in these specific organs due to disruption of the integrity of the blood–brain barrier [93,94] or leaky gut [95,96], both associated with heavy ethanol consumption. Alternatively, ethanol may lead to a down-regulation of arsenic metabolism and clearance [41,97]. Regardless of the mechanism behind the increased levels, a buildup of arsenic in the liver and hippocampus would make the tissue more susceptible to damage. It is important to denote that lower concentrations of ethanol have been purported to increase arsenic absorption but lower accumulation [98]. One limitation of the current study is that only one dose of ethanol was examined as the main objective was to understand alcohol-induced neurodegeneration. Interestingly, the hippocampus appeared to be more sensitive to ethanol’s influence on arsenic content than the rest of the brain. If other metals are also increased by ethanol in a similar pattern [99], the increase in arsenic levels in the hippocampus may indicate a novel mechanism of increased susceptibility associated with the hippocampus compared to other brain regions [6,100]. 

As expected, the level (5 g/kg) and duration (ten days) of ethanol exposure led to neurodegeneration [31,46,101]. Specifically, the current study observed an increase in FJC+ cells following ethanol exposure in all three areas of the hippocampus. The effect of arsenic on FJC+ cell number also aligns with previous studies that examined the neurotoxic effects of arsenic with doses and timelines similar to our ten-day, 10 mg/kg exposure [48,102,103]. However, in the hippocampus, the cell death caused by arsenic alone seemed to be restricted to the DG. There were no synergistic effects at lower doses in the DG, but at the highest dose, there was an increase in FJC+ cells when alcohol and arsenic were combined. 

## 5. Conclusions

Altogether, these studies indicate that the mechanisms of neurodegeneration that are associated with both ethanol and arsenic have slight but significant interactions. In neuroimmune responses, arsenic led to an exacerbated response in microglia and proinflammatory cytokines, particularly in the DG. However, no combinatory effects were observed in ROS/RNS concentrations despite the arsenic-induced increase in CYP2E1. This increase in CYP2E1 may be responsible for the significant differences in BECs that we observed despite consistent doses suggesting that arsenic may impact alcohol pharmacokinetics. The influence on pharmacokinetics was bidirectional as ethanol led to an increase in arsenic in the liver and hippocampus. Figure 7 summarizes our findings and provides potential mechanisms that connect the neurodegenerative effects of alcohol and arsenic co-exposure. It is important to denote that these studies were limited to molecular and cellular determinants that could potentiate neuronal damage; however, an increased proinflammatory microenvironment and neuronal loss in the hippocampus have previously been tied to learning and memory deficits after ethanol [104,105] or arsenic exposure [8,9,49]. Moreover, while both sexes were included in these studies, one major limitation of the current work is that there were not sufficient animal subjects across all experiments to allow determination of whether sex would influence these results. Sex has previously been shown to influence the effects of alcohol on neurodegeneration and neuroimmune responses [56,106,107]. Future studies should consider these limitations examining the mechanistic relationships, sex as a biological variable, and the potential behavioral implications of this alcohol and arsenic co-exposure. 

Despite all these shared mechanisms that were additive and had the capacity to interact with one another (e.g., microglia reactivity increases ROS and ROS can lead to microglial activation), only the DG showed an additive effect when arsenic and ethanol were combined. The ability of chronic alcohol exposure and heavy arsenic exposure to cause neurodegeneration independently was previously established, but these data suggest that the combined toxicity of the two could exacerbate damage in the brain and liver. This work has implications for individuals who are exposed to higher arsenic levels as well as for individuals with AUDs. Altogether, these studies provide evidence that excessive alcohol can worsen the accumulation of arsenic, and likewise, arsenic can enhance the maladaptations in the CNS associated with a history of chronic alcohol use. 

## Figures and Tables

**Table 1:** Experimental Group Descriptions. ♀ = female ♂ = male.

Experiment	Treatment	No Arsenic	0.005 mg/kg	2.5 mg/kg	10 mg/kg
**Immunohistochemistry**	Ethanol	*n* = 8 ♀ = 4; ♂ = 4	*n* = 8 ♀ = 4; ♂ = 4	*n* = 9 ♀ = 4; ♂ = 5	*n* = 8 ♀ = 5; ♂ = 3
	Water	*n* = 8 ♀ = 5; ♂ = 3	*n* = 7 ♀ = 4; ♂ = 4	*n* = 8 ♀ = 3; ♂ = 5	*n* = 10 ♀ = 5; ♂ = 5
**ELISA and ROS Assay**	Ethanol	*n* = 6 ♀ = 3; ♂ = 3	-	-	*n* = 8 ♀ = 4; ♂ = 4
	Water	*n* = 6 ♀ = 3; ♂ = 3	-	-	*n* = 8 ♀ = 4; ♂ = 4
**ICP-MS**	Ethanol	*n* = 12 ♀ = 6; ♂ = 6	-	-	*n* = 14 ♀ = 7; ♂ = 7
	Water	-	-	-	*n* = 14 ♀ = 7; ♂ = 7

**Table 2:** Percent Change in Body Weights. No significant differences were determined between the treatment groups in changes in body weights within any of the cohorts. Immunohistochemistry (Interaction: [F (3, 58) = 0.59, *p* = 0.63]; Ethanol [F (1, 58) = 0.14, *p* = 0.71]; Arsenic [F (3, 58) = 0.48, *p* = 0.69]); ELISA/ROS (Interaction: [F (1, 24) = 0.29, *p* = 0.59]; Ethanol [F (1, 24) = 0.10, *p* = 0.75]; Arsenic [F (1, 24) = 2.6, *p* = 0.12]); ICP-MS [F (2, 37) = 0.13, *p* = 0.88].

Experiment	Treatment	No Arsenic	0.005 mg/kg	2.5 mg/kg	10 mg/kg
**Immunohistochemistry**	Ethanol	µ = 0.4 ± 0.3	µ = −0.8 ± 1.7	µ = 0.4 ± 1.5	µ = 0.9 ± 2.5
	Water	µ = 1.8 ± 1.5	µ = 1.1 ± 1.1	µ = −1.4 ± 1.1	µ = 0.9 ± 1.3
**ELISA and ROS Assay**	Ethanol	µ = 1.9 ± 2.6	-	-	µ = −1.6 ± 1.7
	Water	µ = 1.6 ± 1.2	-	-	µ = −0.2 ± 0.8
**ICP-MS**	Ethanol	µ = −1.6 ± 1.2	-	-	µ = 1.0 ± 0.9
	Water	-	-	-	µ = −0.9 ± 1.2

## Data Availability

The data presented in this study are available on request from the corresponding author.
